# CENP-A Ubiquitylation Contributes to Maintaining the Chromosomal Location of the Centromere

**DOI:** 10.3390/molecules24030402

**Published:** 2019-01-22

**Authors:** Yohei Niikura, Risa Kitagawa, Katsumi Kitagawa

**Affiliations:** 1 Greehey Children’s Cancer Research Institute, Department of Molecular Medicine, UT Health Science Center San Antonio School of Medicine, 8403 Floyd Curl Drive, San Antonio, TX 78229-3000, USA; kitagawaR@uthscsa.edu; 2 MOE Key Laboratory of Model Animal for Disease Study, Model Animal Research Center, Nanjing University, Nanjing 210061, China

**Keywords:** CENP-A, centromere identity, centromere, CUL4A-RBX1- COPS8 E3 ligase, dimerization, epigenetics, kinetochore, lysine 124 (K124), neocentromere, ubiquitylation

## Abstract

The centromere plays an essential role in accurate chromosome segregation, and the chromosomal location of the centromere is determined by the presence of a histone H3 variant, centromere protein A (CENP-A), in centromeric nucleosomes. However, the precise mechanisms of deposition, maintenance, and inheritance of CENP-A at centromeres are unclear. We have reported that CENP-A deposition requires ubiquitylation of CENP-A lysine 124 mediated by the E3 ligase activity of Cullin 4A (CUL4A)—RING-box protein 1 (RBX1)—COP9 signalsome complex subunit 8 (COPS8). We have proposed a model of inheritance for CENP-A ubiquitylation, through dimerization between rounds of cell divisions, that maintains the position of centromeres.

## Main Text

During cell division, proper chromosomal segregation must be achieved; otherwise chromosomes may be unequally distributed among daughter cells. In most eukaryotes, the centromere is a highly constricted single region of a chromosome where the spindle microtubules attach during cell division (mitosis and meiosis). The major role of the centromere is to provide sites for the kinetochore, the protein complexes that bind microtubules of the spindle bundle during cell division. The most inner components of the kinetochore consist of the electron dense plate that is juxtaposed to the centromeric chromatin as observed in electron microscope [1]. The temporal-spatial regulation and structures of centromere and kinetochore proteins are important keys to understand chromosome instability (CIN) that results in aneuploidy (i.e., an incorrect number of chromosomes). The causes and the consequences of aneuploidy are poorly understood, although aneuploidy usually results in tumorigenesis, birth defects, and developmental disorders such as Down syndrome.

Therefore, there are several fundamental questions to be solved: What mechanism determines the specific chromosomal location of the functional centromere (i.e., centromere identity)? How are they regulated during accurate chromosome segregation?

In most species except budding yeast [2], the centromere has no defined DNA sequence but consists of large arrays of repetitive DNA, and centromeric DNA is not sufficient or necessary to define centromere identity. Centromere identity is defined in an epigenetic manner by the presence of a special nucleosome that contains a centromere-specific histone H3 variant, centromere protein A (CENP-A). Proper deposition of CENP-A at the centromere is required for proper centromere inheritance and function, and this epigenetic mechanism is a fundamental biological process. Nucleosomes that contain CENP-A (a.k.a., CENP-A nucleosomes) localize to the inner kinetochores. Such CENP-A nucleosomes consist of the canonical histones H2A, H2B, and H4 at the active centromeres. CENP-A nucleosomes and are required for active centromeres to recruit a constitutive centromere-associated network (CCAN) and the other kinetochore proteins [2]. Therefore, although CENP-A is proposed as the non-DNA indicator (epigenetic marker) of centromere identity, the key question remains how CENP-A defines the position of the centromere in humans. We have reported that E3 ligase activity of CUL4A (Cullin 4A)-RBX1 (Ring-box 1)-COPS8 (COP9 signalosome complex subunit 8) is required for ubiquitylation of lysine 124 (K124) in CENP-A and centromere localization of CENP-A during the M and G1 phases [3]. Recently, we suggested a model of inheritance of CENP-A ubiquitylation to regulate CENP-A localization and maintenance at centromeres [4]. In this model, CENP-A K124 ubiquitylation is inherited in an epigenetic manner through dimerization between different rounds of cell divisions.

**Figure 1:**
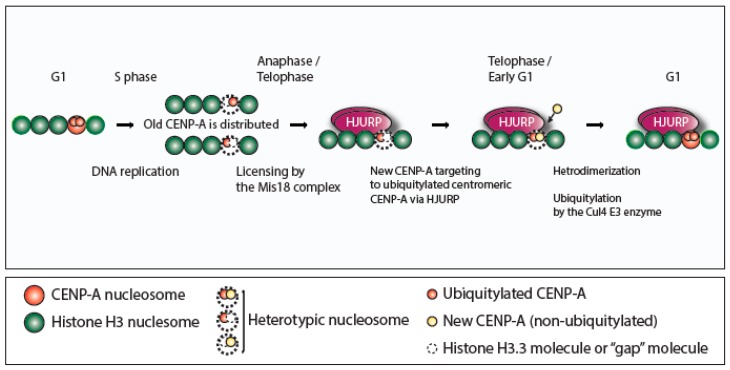
Model of epigenetic inheritance of centromere protein A (CENP-A) ubiquitylation.

We demonstrated that ubiquitylated CENP-A is required for ubiquitylation of nonubiquitylated CENP-A in our in vivo and in vitro assays using constitutively ubiquitylated CENP-A. Therefore, we suggest that the heterodimer (i.e., a dimer of old CENP-A and new CENP-A) is presumably recognized by the CUL4A complex, and the new CENP-A is ubiquitylated and maintained at the centromeres [3]. CENP-A–containing nucleosomes are formed with the canonical histones H2A, H2B, and H4 at the active centromeres. However, it is still controversial whether the interconversion between tetrameric and octameric CENP-A nucleosomes in the cell cycle is critical [2]. Therefore, here we adapt a proposed octamer model of epigenetic inheritance of CENP-A ubiquitylation (Figure 1) as previously discussed [5].

In this tentative octamer model, two CENP-A dimers in one nucleosome are distributed separately between two daughter centromere-DNA sequences, and one CENP-A molecule either is exchanged with one H3 molecule or leaves a molecule-free gap during the replication/S phase. HJURP (Holliday junction recognition protein) preferentially binds to ubiquitylated, preassembled “old” CENP-A, which is situated predominantly in nucleosomes. A new CENP-A monomer targets ubiquitylated centromeric CENP-A via preassembled HJURP. New CENP-A is properly ubiquitylated in a heterodimerization-dependent manner (i.e., dimers of old CENP-A with new CENP-A). In this way, the ubiquitylation and the location of the centromere are inherited epigenetically. Note that histone H4 is omitted for simplicity.

Starting at DNA replication, this tentative octamer model proposes that two pre-assembled “old” CENP-A molecules in one nucleosome are distributed separately between two daughter centromere-DNA sequences (Figure 1, S phase). One CENP-A molecule is either replaced with one histone H3.3 molecule or absent, leaving a molecule-free place (“gap”) during replication/S phase (Figure 1, S phase). This idea is supported by the previous report that histone H3.3 is deposited at centromeres during the S phase and occupied as a placeholder for CENP-A that is newly deposited and/or replaced with histone H3.3 during the G1 phase [6] (Figure 1, S phase).

A protein called HJURP (Holliday junction recognition protein) is a specific chaperone protein of CENP-A with nucleosome assembly activity specifically for newly synthesized CENP-A [7,8,9]. The evidence from our studies and others [4] supports our concept (Figure 1) that HJURP preferentially binds to preassembled “old” CENP-A, which has a ubiquitin group and is situated predominantly in nucleosomes to initiate the ubiquitylation of newly synthesized (‘‘new’’) CENP-A (Figure 1, anaphase/telophase). Pre-assembled ubiquitylated centromeric CENP-A in nucleosomes is targeted by newly synthesized, free CENP-A through its interaction to HJURP (Figure 1, telophase/early G1). In a heterodimerization-dependent manner (through interaction of old CENP-A – new CENP-A), new CENP-A is ubiquitylated near the nucleosome and/or inside the nucleosomes (Figure 1, telophase/early G1). Our results showed that HJURP partly contributes to ubiquitylation, because addition of purified HJURP protein enhanced the CENP-A ubiquitylation in vitro, and HJURP siRNA led to a significant reduction of the CENP-A ubiquitylation in vivo. Thus, ubiquitylation and the location of the centromere are inherited in an epigenetic manner through dimerization between different rounds of mitosis (Figure 1).

Neocentromeres are ectopic sites on chromosomes where new functional kinetochores assemble to specify and conduct chromosome segregation. Over 100 neocentromeres have been described in clinical samples [10]. However, they form on very diverse DNA sequences not associated with alpha-satellite DNA. These findings verified that the formation of human centromeres does not depend on primary DNA sequences, and centromeres are inherited in an epigenetic manner. However, the mechanism to create human neocentromeres is not yet clear; mere overexpression of CENP-A results in mislocalization of CENP-A but not the successful formation of functional neocentromeres [11]. Therefore, it is important to investigate factors required for generation of human neocentromeres to elucidate the mechanism of epigenetic inheritance of centromeres. We constructed an N-terminal Flag-tagged and C-terminal ubiquitin-fused K124R CENP-A mutant, and in this construct, we also applied the monoubiquitin mutant Ub (K48R), which has lost a major site for polyubiquitylation to prevent ubiquitin-fused CENP-A protein from potential polyubiquitylation. We found that overexpression of the monoubiquitin fusion protein Flag-CENP-A K124R-Ub (K48R) sufficiently recruited HJURP and central-outer kinetochore components to ectopic chromatin [4]. In particular, putative neocentromeres, where SKA1 was properly recruited, were replicated and inherited epigenetically between cell division.

Many studies have proposed that CENP-A is the epigenetic marker of the centromere identity [2]. However, we have shown that overexpression of CENP-A itself is not sufficient to generate a neocentromere (Figure 1) and that ubiquitylation of CENP-A is required for neocentromere formation or epigenetic inheritance of the centromere location in humans [4]. We conclude that CENP-A ubiquitylation is a candidate as an epigenetic marker of centromere location, (i.e., centromere identity), considering that histone posttranslational modifications are traditionally defined as “epigenetic markers”.

Our studies were performed using the HeLa cervical carcinoma cells in which the p53 and pRB signaling pathways are disrupted due to Human papillomavirus (HPV) 18 E6 infection. Filipescu et al. suggested the essential role of CENP-A and its specific chaperone HJURP following p53 loss in tumor progression in both in vitro and in vivo experiments [12]. They demonstrated that functional p53 elicits a cell cycle arrest response, whereas, in p53-null transformed cells, the absence of arrest enables the loss of HJURP to induce severe aneuploidy and apoptotic cell death, discussing a model of “epigenetic addiction” in which the rapidly proliferating cells in p53-null tumors become highly dependent on the HJURP [12]. To further establish the generality of our model it would be interesting to study how transcriptional and/or post-transcriptional regulation of CENP-A and HJURP through p53 is functionally involved in our model.

Overexpression of CENP-A and formation of neocentromere to a chromosome make the chromosome very unstable, and this instability results in aneuploidy and possibly in cancer generation [13,14,15]. Sun et al. demonstrated that elevated CENP-A expression can be used as a prognostic and predictive cancer biomarker, especially for taxane-based chemotherapy and possibly other treatments targeting cell division, using genomic, transcriptomic and patient information from databases [16]. Lacoste et al. suggested that CENP-A overexpression in human cells leads to ectopic localization at the CTCF binding sites with active histone turnover involving a heterotypic tetramer containing CENP-A-H4 with H3.3-H4 [13]. Solving the composition and dynamics of centromeric and non-centromeric nucleosomes is one of the intriguing directions of the future research. Thus, it is essential to understand the mechanism that controls the amount of CENP-A, including stoichiometry of CENP-A nucleosomes, to elucidate the mechanism of neocentromere formation.

## References

[1] Saitoh H., Tomkiel J., Cooke C.A., Ratrie H., Maurer M., Rothfield N.F., Earnshaw W.C. (1992). CENP-C, an autoantigen in scleroderma, is a component of the human inner kinetochore plate. Cell.

[2] Fukagawa T., Earnshaw W.C. (2014). The centromere: Chromatin foundation for the kinetochore machinery. Dev. Cell.

[3] Niikura Y., Kitagawa R., Ogi H., Abdulle R., Pagala V., Kitagawa K. (2015). CENP-A K124 Ubiquitylation Is Required for CENP-A Deposition at the Centromere. Dev. Cell.

[4] Niikura Y., Kitagawa R., Kitagawa K. (2016). CENP-A Ubiquitylation Is Inherited through Dimerization between Cell Divisions. Cell Rep..

[5] Niikura Y., Kitagawa R., Kitagawa K. (2016). The inheritance of centromere identity. Mol Cell Oncol..

[6] Dunleavy E.M., Almouzni G., Karpen G.H. (2011). H3.3 is deposited at centromeres in S phase as a placeholder for newly assembled CENP-A in G(1) phase. Nucleus.

[7] Foltz D.R., Jansen L.E., Bailey A.O., Yates J.R., Bassett E.A., Wood S., Black B.E., Cleveland D.W. (2009). Centromere-specific assembly of CENP-a nucleosomes is mediated by HJURP. Cell.

[8] Dunleavy E.M., Roche D., Tagami H., Lacoste N., Ray-Gallet D., Nakamura Y., Daigo Y., Nakatani Y., Almouzni-Pettinotti G. (2009). HJURP is a cell-cycle-dependent maintenance and deposition factor of CENP-A at centromeres. Cell.

[9] Barnhart M.C., Kuich P.H., Stellfox M.E., Ward J.A., Bassett E.A., Black B.E., Foltz D.R. (2011). HJURP is a CENP-A chromatin assembly factor sufficient to form a functional de novo kinetochore. J. Cell Biol..

[10] Marshall O.J., Chueh A.C., Wong L.H., Choo K.H. (2008). Neocentromeres: New insights into centromere structure, disease development, and karyotype evolution. Am. J. Hum. Genet..

[11] Van Hooser A.A., Ouspenski I.I., Gregson H.C., Starr D.A., Yen T.J., Goldberg M.L., Yokomori K., Earnshaw W.C., Sullivan K.F., Brinkley B.R. (2001). Specification of kinetochore-forming chromatin by the histone H3 variant CENP-A. J. Cell Sci..

[12] Filipescu D., Naughtin M., Podsypanina K., Lejour V., Wilson L., Gurard-Levin Z.A., Orsi G.A., Simeonova I., Toufektchan E., Attardi L.D. (2017). Essential role for centromeric factors following p53 loss and oncogenic transformation. Genes Dev..

[13] Lacoste N., Woolfe A., Tachiwana H., Garea A.V., Barth T., Cantaloube S., Kurumizaka H., Imhof A., Almouzni G. (2014). Mislocalization of the centromeric histone variant CenH3/CENP-A in human cells depends on the chaperone DAXX. Mol. Cell..

[14] Tomonaga T., Matsushita K., Yamaguchi S., Oohashi T., Shimada H., Ochiai T., Yoda K., Nomura F. (2003). Overexpression and mistargeting of centromere protein-A in human primary colorectal cancer. Cancer Res..

[15] Gascoigne K.E., Cheeseman I.M. (2013). Induced dicentric chromosome formation promotes genomic rearrangements and tumorigenesis. Chrom. Res..

[16] Sun X., Clermont P.L., Jiao W., Helgason C.D., Gout P.W., Wang Y., Qu S. (2016). Elevated expression of the centromere protein-A(CENP-A)-encoding gene as a prognostic and predictive biomarker in human cancers. Int. J. Cancer.

